# A Case of Acute Cerebral Infarction With Chief Complaints of Abdominal Pain and Bloody Diarrhoea: The Power of a Patient-Centered Inclusive Diagnostic Team

**DOI:** 10.7759/cureus.27386

**Published:** 2022-07-28

**Authors:** Taichi Fujimori, Tsunetaka Kijima, Satoshi Honda, Shingo Yamagata, Tetsuya Makiishi

**Affiliations:** 1 Internal Medicine, Shimane University, Izumo, JPN; 2 General Medicine, Shimane University, Izumo, JPN; 3 Nephrology, Shimane University, Izumo, JPN; 4 Neurology, Shimane University, Izumo, JPN

**Keywords:** early closure, cognitive error, bias, heuristic, diagnostic team, diagnostic error

## Abstract

We present the case of a 66-year-old man who presented with acute abdominal pain and bloody stool as his chief complaints and was finally diagnosed with ischemic colitis from colon cancer and acute cerebral infarction. Although several cognitive biases led to physicians missing the presence of acute stroke, a diagnostic team consisting of the patient, his family members, a ward nurse, and the physician worked effectively to reach the correct diagnosis soon after admission. A physician is not the only person involved in the diagnostic process. A patient-centered diagnostic team is necessary.

## Introduction

It is expected that all patients will be accurately diagnosed in daily clinical practice. However, this is not always achieved in actual medical practice, simply because most clinical diagnoses are currently made by humans. A recent study indicated that a harmful diagnostic error occurs in 0.7% (95% confidence interval, 0.5%-1.1%) of hospitalized adults [1]. In Japan, this estimate corresponds to approximately 9,200 harmful diagnostic errors annually [2], making it a serious public health burden. 

To address this issue, multiple measures have been proposed, most of which focus on physicians and healthcare systems with limited success [3,4]. Recently, the concept has been introduced that an inclusive diagnostic team (sometimes called 'effective diagnostic team') consists of the patient and their family, the physician, and associated medical staff and has become essential in the diagnostic process [5,6]. Although the phrase 'diagnostic team' has been used in the literature since the 1960s [7], it has focused on the idea that diagnostic processes in collaboration with a specialist for each field could be more accurate [8,9]. In contrast, a landmark report published by the National Academy of Medicine in 2015 emphasized the dynamics of the diagnostic process, which is carried out by a patient-centered diagnostic team, beyond just the collection of knowledge and skills of specialists [5]. The report forecasted that successful diagnosis in the 21st century will become a team-based activity, which leverages the knowledge and skills of all interprofessional staff involved in the case and will involve the patient and their family members as active team members [5]. This report focused on 'effective teamwork' as its first recommendation [5]. No one knows their medical history and symptoms better than the patients and their families. They are important contributors to the diagnostic team [10]. Nurses are now recognized as active members of the diagnostic process because of their unique relationship with both the patient and physician [11]. In addition, good teamwork is not only useful for patient safety but also for the well-being and satisfaction of healthcare professionals [12].

In this case, the patient presented to our emergency room (ER) complaining of abdominal pain. The physician's initial diagnosis of ischemic colitis was correct. However, the physician missed another pathology. A diagnostic team collaborated effectively to reach the second correct diagnosis.

## Case presentation

A 66-year-old man presented to the ER at our hospital complaining of acute lower abdominal pain that had started two hours prior, and bloody stools for a month. He reported a similar episode of abdominal pain occurring a month ago. Although the patient was previously diagnosed with hypertension, dyslipidemia, and liver dysfunction at a medical checkup a year ago, he had never received medical care for these problems.

His level of consciousness at the ER was not fully lucid. The physician in charge believed that the patient's mental status was mildly abnormal, noting the consciousness level in the medical records as Japan Coma Scale (JCS) I-1 (mostly clear, but not perfect). Physical examination revealed tenderness in the central hypogastric region. There was no obvious motor paralysis or decreased sensation in the limbs or face.

**Figure 1:**
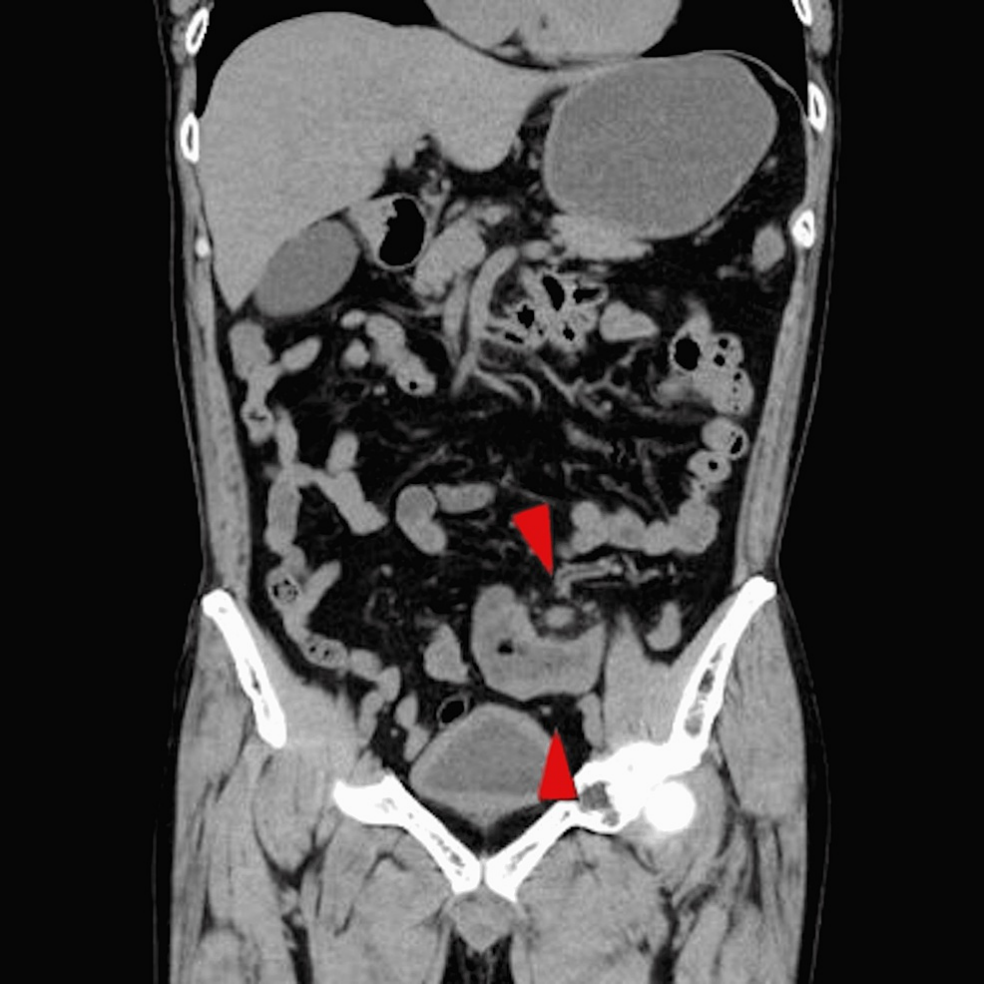
Non-contrast abdominal CT (coronal plane) showing edematous thickening of the wall extending the left side of the transverse colon to the sigmoid colon, enlarged lymph nodes, and increased density in the fatty tissue of the surrounding mesentery (arrows)

Representative laboratory test results were as follows: hemoglobin 13.2 g/dL; white blood cells 13,630 /μL; platelets 385×103/μL; and C-reactive protein 1.0 mg/dL. Non-contrast abdominal computed tomography (CT) revealed edematous thickening of the colon wall, extending from the left side of the transverse colon to the sigmoid colon. It also revealed several enlarged lymph nodes and increased density in the fatty tissue of the surrounding mesentery (Figure 1). The physician diagnosed the patient with ischemic colitis and admitted him to the hospital for further investigation and treatment.

**Figure 2:**
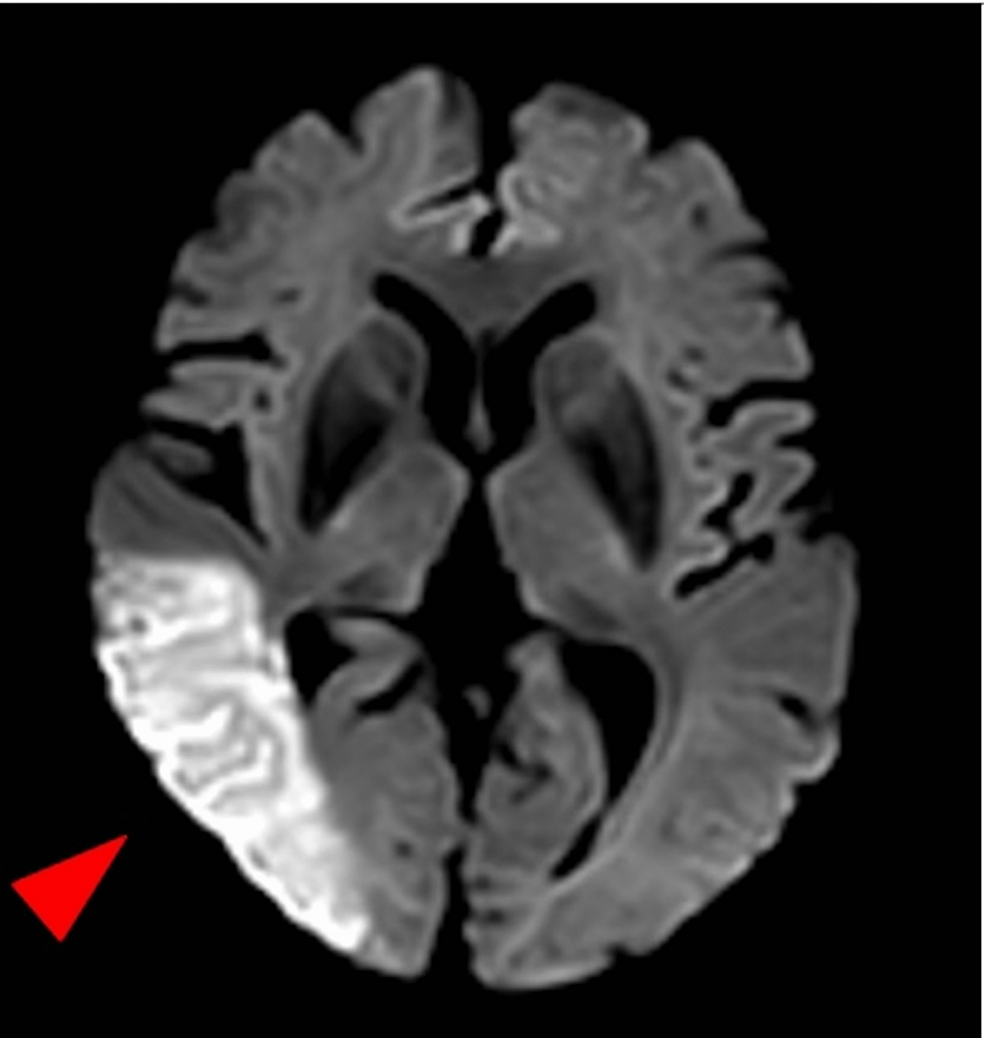
Head MRI showing high intensity in the right occipital lobe on diffusion-weighted imaging (arrow)

A few hours after admission, his family members (wife and daughter) and a ward nurse noticed that “something was wrong” with the patient’s attitude and suggested that the physician re-examine the patient. Further history-taking revealed that the patient had experienced some difficulty with his vision just before he visited the ER. Detailed neurological examination revealed diplopia, insufficient convergence, left visuospatial neglect, auditory extinction in the left ear, and decreased sensation in the left upper limb. At this time, acute cerebral infarction was suspected and confirmed following head magnetic resonance imaging (MRI) (Figure 2). The etiology was thought to be artery-to-artery embolism, given the MRI and contrast-enhanced cervical CT showing 25% stenosis of the right internal carotid artery.

Aspirin and ozagrel (a thromboxane synthase inhibitor) were started on the first day of admission and discontinued on the fourth day when the follow-up head MRI revealed hemorrhage around the infarcted area. On the 16th day, considering that embolic cerebral infarction was the more likely etiology, we couldn’t identify conclusive evidence about the cause of acute cerebral infarction, and edoxaban, a Xa inhibitor, was initiated instead of aspirin and ozagrel.

Two weeks after admission, a colonoscopy was performed, which revealed a large colorectal cancer that was difficult for the endoscope to pass through, and a stent was placed to cover the lesion. Laparoscopic sigmoid colectomy was postponed approximately 70 days after admission to avoid relapse of the cerebral infarction and hemorrhage. According to the tumor, node, metastasis (TNM) staging system, the stage of his tumor was cT4N1bM0, stage IIIb. Chemotherapy with oxaliplatin was planned for outpatient treatment. Eighty-five days after admission, the patient was discharged.

## Discussion

Diagnostic errors can occur in daily clinical practice; we need to be aware of them and embrace effective measures to prevent them. In this case, the patient had two life-threatening diseases at the time of visiting the ER: ischemic enteritis because of colorectal cancer [13], and cerebral infarction. Although several cognitive biases such as availability heuristic, early closure, and anchoring heuristic interfered with the physician’s clinical reasoning in the ER [14], a diagnostic team consisting of the patient, his family, and a ward nurse, in addition to the physician in charge, worked effectively to correct the initial faulty clinical reasoning of the physician alone.

Recently, a new concept of an 'inclusive diagnostic team' has been proposed, insisting that the diagnostic process should be shared with and made by an inclusive diagnostic team consisting of the patient and their family, the physician, and associated medical staff [5,6].

Our hospital had not implemented the new concept of 'an inclusive diagnostic team' in daily clinical practice until the reported case. However, the fact that we reached the correct diagnosis of this case, although delayed by a couple of hours, might show the potential for the concept exists among the medical staff involved in the case. Good teamwork depends on communication skills (timely, frequent, accurate, and effective) and role relationships (shared goals, shared knowledge, and mutual respect) [15]. Especially now, electronic health records and other modern health information technologies have created a lack of face-to-face communication among healthcare professionals, leading to diagnostic errors [16]. In this case, the nurse conveyed his impressions directly to the physician, and the physician accepted suggestions from the nurse and the patient’s family. At a minimum, there was mutual respect for listening and being heard among them.

Unfortunately, this concept of an inclusive diagnostic team has not been fully accepted in daily clinical practice, where traditional diagnostic processes still prevail. The lack of attention to this concept is partly evidenced by the lack of case reports in this area. A one-size-fits-all model of an inclusive diagnostic team does not exist [6]. Each organization needs to make efforts to improve teamwork according to its unique assets and structure. Among such efforts, outcome feedback to the medical staff involved in each case is essential to promote learning and improvement related to diagnosis [17]. We began by sharing this case with other medical staff at our hospital. We have also tried to foster an atmosphere in multidisciplinary meetings where all members feel they can contribute to discussions [18]. Most importantly, everyone working at the healthcare institution recognizes the concept of a patient-centered inclusive diagnostic team. Continuous efforts are needed to expand this idea.

## Conclusions

We report a case of colon cancer and acute cerebral infarction in a patient who presented with abdominal pain and bloody diarrhea. Although several cognitive biases such as ”premature closure” led physicians to miss the presence of acute cerebral infarction, a diagnostic team worked effectively to reach the correct diagnosis soon after admission. Physicians are not the only healthcare professionals involved in the diagnostic process. A patient-centered inclusive diagnostic team is the best approach to making all the correct diagnoses in the modern era when most adult patients have multiple comorbidities. We, healthcare professionals, need to report such cases, emphasize the importance of the diagnostic team, and work with other healthcare professionals to decrease diagnostic errors.
